# ROS-induced oxidative stress in plant cryopreservation: occurrence and alleviation

**DOI:** 10.1007/s00425-021-03784-0

**Published:** 2021-11-20

**Authors:** Li Ren, Min-Rui Wang, Qiao-Chun Wang

**Affiliations:** 1 Institute for Agri-Food Standards and Testing Technology, Shanghai Academy of Agricultural Sciences, Shanghai, 201403 People’s Republic of China; 2 State Key Laboratory of Crop Stress Biology for Arid Region, College of Life Science, Northwest A&F University, Yangling, 712100 Shaanxi People’s Republic of China; 3 State Key Laboratory of Crop Stress Biology for Arid Region, College of Horticulture, Northwest A&F University, Yangling, 712100 Shaanxi People’s Republic of China

**Keywords:** Antioxidants, Cryopreservation, Gene expression, Oxidative stress, Programmed cell death, Reactive oxygen species

## Abstract

**Main conclusion:**

Reactive oxygen species (ROS)-induced oxidative stress results in low success or even total failure of cryopreservation. Better understanding of how the plant establishes resistance/tolerance to ROS-induced oxidative stress facilitates developments of robust cryopreservation procedures.

**Abstract:**

Cryopreservation provides a safe and efficient strategy for long-term preservation of plant genetic resources. ROS-induced oxidative stress caused damage to cells and reduced the ability of the plant to survive following cryopreservation, eventually resulting in low success or even total failure. This paper provides updated and comprehensive information obtained in the past decade, including the following: (1) ROS generations and adaptive responses of antioxidant systems during cryopreservation; (2) expressions of oxidative stress-associated genes and proteins during cryopreservation; (3) ROS-triggered programmed cell death (PCD) during cryopreservation; and (4) exogenous applications of enzymatic and non-enzymatic antioxidants in improving success of cryopreservation. Prospects for further studies are proposed. The goal of the present study was to facilitate better understanding of the mechanisms by which the plant establishes resistance/tolerance to oxidative stress during cryopreservation and promote further studies toward the developments of robust cryopreservation procedures and wider application of plant cryobiotechnology.

## Introduction

Plant cryopreservation refers to storage of plant cells, tissue and organs in liquid nitrogen (LN, − 196 °C) or liquid nitrogen vapor (LNV, approx. − 165 to − 190 °C) (Kaczmarczyk et al. 2012; Wang et al. 2021a). Cryopreservation is considered at present time an ideal means for long-term preservation of plant genetic resources (Kaczmarczyk et al. 2012; Bettoni et al. 2021; Wang et al. 2014, 2021a, b). Cryo-banks using shoot tips have been set up in several countries for economically important plant species (Kulus and Zalewska 2014; Wang et al. 2014; Vollmer et al. 2016; Jenderek and Reed 2017). Cryobiotechnology has also been extended to long-term preservation of transgenes in transgenic cells (Wang et al. 2012, 2014), production of pathogen-free plants (Wang and Valkonen 2009; Wang et al. 2014; Zhao et al. 2019) and long-term preservation of obligate pathogens such as viruses and viroids (Zhao et al. 2019). Studies have also advanced in the evaluation of the performance of cryo-derived plants when they were re-introduced from laboratories to their natural habitats (Salama et al. 2018; Bi et al. 2021). However, ROS-induced oxidative stress is recognized to be a major constraint for further developments of plant cryopreservation, particularly endangered, endemic and tropical species, which are still recalcitrant to cryopreservation (Kaczmarczyk et al. 2012; Reed 2014; Funnekotter et al. 2017; Normah et al. 2019; Streczynski et al. 2019; Coelho et al. 2020).

As signaling molecules, ROS supports cellular proliferation and physiological function, and therefore, maintenance of a basal level of ROS is essential for life (Mittler 2017). However, overproduction of ROS is highly reactive and toxic and causes damage to DNA and protein, as well as membrane oxidation (Gill and Tuteja 2010; Mittler 2017). Under normal circumstances, there is a balance between ROS production and clearance in plants. However, under stress conditions, excessive ROS are produced, resulting in oxidative stress (Gill and Tuteja 2010). Oxidative stress refers to a state of imbalance between oxidation and anti-oxidation, which is caused by excessive generations of ROS (Halliwell 2006; Mittler 2017). O_2_⋅−, H_2_O_2_, and OH⋅ are the three major ROS and H_2_O_2_ is the main ROS component that causes membrane lipid peroxidation in plant cryopreservation (Chen et al. 2015; Ren et al. 2015; Zhang et al. 2015). Detailed information on generation of O_2_⋅−, H_2_O_2_ and OH⋅ can be found in the articles of Mittler (2002) and Vranová et al. (2002).

In response to oxidative stress, plants develop antioxidant defense systems via enzymatic and non-enzymatic antioxidant reactions to maintain normal metabolisms and functions in the cell, thus protecting themselves against oxidative stress (Gill and Tuteja 2010). An antioxidant is defined as a molecule or compound that can delay, prevent, or remove damage caused by oxidative stress (Halliwell 2006). Plant antioxidants include two types: enzymatic and non-enzymatic, both of which can regulate ROS production (Halder et al. 2018). Enzymatic antioxidants mainly include superoxide dismutase (SOD), peroxidase (POD), catalase (CAT), ascorbate peroxidase (APX), monodehydroascorbate reductase (MDHAR), glutathione reductase (GR), glutathione peroxidase (GPX) and dehydroascorbate reductase (DHAR) (Gill and Tuteja 2010). Non-enzymatic antioxidants consist of many members, for example, glutathione (GSH), ascorbic acid (AsA), abscisic acid (ABA), tocopherol (VE) and melatonin (Gill and Tuteja 2010; Nawaz et al. 2016; Sah et al. 2016). Among these antioxidants, SOD functions in scavenging O_2_⋅− and lowering the possibility of OH⋅ production (Scandalios 1990). POD, CAT and APX play an essential protective role in scavenging H_2_O_2_ when coordinating with SOD (Chaitanya et al. 2002). Melatonin fortifies plants against abiotic and biotic stress, mainly by scavenging ROS and reactive nitrogen species (Nawaz et al. 2016). AsA is the most effective water-soluble antioxidant in plants, which can provide electrons to a large number of antioxidants (Gill and Tuteja 2010) and scavenge O_2_⋅− and H_2_O_2_ (Noctor and Foyer 1998; Smirnoff 2000). GSH helps to scavenge ROS via AsA-GSH cycle and a balance between GSH and oxidized glutathione is important for maintaining the redox status of cells (Pastori et al. 2000; Gill and Tuteja 2010).

**Fig. 1:**
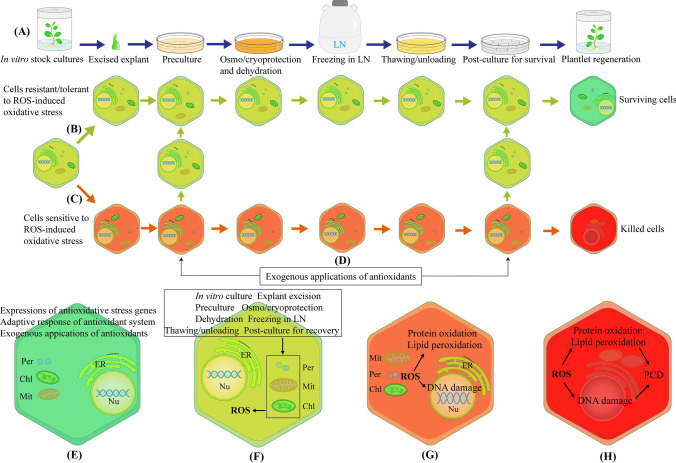
Hypothetical illustration of ROS-induced oxidative stress, oxidative stress-caused cell damage and PCD, adaptive responses of antioxidant system, expressions of antioxidative stress genes, and exogenous applications of antioxidants for improving recovery of cryopreserved shoot tips. **A** Major steps of cryopreservation procedure. **B** Responses of cells that are resistant/tolerant to oxidative stress by expressions of antioxidative genes and proteins to alleviate the oxidative stress during cryopreservation, thus helping cells to survive after cryopreservation. **C** Responses of cells that are sensitive to oxidative stress during cryopreservation. Oxidative stress causes membrane lipid peroxidation, protein oxidation and DNA damage, and induced programmed cell death (PCD), eventually killing the cells after cryopreservation. **D** Exogenous applications of antioxidants to preculture and/or post-culture media for improving recovery of cryopreserved plants. **E** A representative cell (green color) that is resistant/tolerant to oxidative stress, in which antioxidative stress genes are expressed, and adaptive responses of antioxidant system are established to respond to oxidative stress. Exogenous applications of enzymatic and non-enzymatic antioxidants alleviate ROS-induced oxidative stress and improve recovery of cryopreserved plants. Such cells are most likely to survive after cryopreservation. **F** A representative cell (yellow color), in which ROS generation is induced in in vitro culture and the major steps of cryopreservation procedure. **G** A representative cell (light red color) that is sensitive to oxidative stress, in which ROS causes protein oxidation, lipid peroxidation and DNA damage. **H** A representative cell (red color) that is sensitive to oxidative stress, in which PCD is induced. *Chl* chloroplasts, *ER* endoplasmic reticulum, *Mit* mitochondria, *Nu* nucleus, *PCD* programmed cell death, *Per* peroxisomes, *ROS* reactive oxygen species

Plant cryopreservation requires several necessary steps such as the establishment of stock cultures, excision of explants, preculture, osmoprotection and cryoprotection, dehydration, freeze–thaw cycle, unloading and post-culture for recovery (Fig. 1A). All these steps induce ROS generation (Fig. 1B, C, E, F). ROS-induced oxidative stress reduces the ability of the explant to survive following cryopreservation, eventually resulting in poor recovery or even total failure (Benson and Bremner 2004; Kaczmarczyk et al. 2012; Reed 2014; Funnekotter et al. 2017; Normah et al. 2019; Streczynski et al. 2019; Coelho et al. 2020; Fig. 1B, C, F–H).

This review focuses on advances over the past decade in analyses of ROS generation and adaptive responses of the antioxidant system during cryopreservation procedures. ROS-induced programmed cell death (PCD), and expressions of oxidative stress-associated genes and proteins in cryopreservation are also presented. Finally, applications of enzymatic and non-enzymatic antioxidants to improvements of recovery of cryopreserved explants are presented in detail.

## ROS generation, programmed cell death (PCD) and adaptive responses of antioxidant system in plant cryopreservation

### ROS generation

In vitro tissue culture has become an integral part of the cryopreservation technology currently used for the establishment and maintenance of stock cultures, and the post-culture process for the recovery of cryopreserved tissues (Funnekotter et al. 2017; Wang et al. 2021b; Fig. 1A). It is well documented that in vitro tissue culture imposes stressful conditions and induces the generations of ROS, thus resulting in ROS-induced oxidative stress (Kaczmarczyk et al. 2012; Funnekotter et al. 2017; Bednarek and Orłowska 2020; Wang et al. 2021b; Fig. 1B, F). Cold-hardening of the in vitro stock cultures was frequently used for improving plant cryopreservation (Kaczmarczyk et al. 2012; Funnekotter et al. 2017). Cold-hardening of the in vitro stock cultures was proven to induce expressions of antioxidant enzymes, such as SOD and catalase, increase antioxidant levels, and maintain membrane stability, eventually enhancing recovery of cryopreserved plants (Kaczmarczyk et al. 2012; Funnekotter et al. 2017).

It has been known for a long time that age of stock cultures from which explants were excised and used for cryopreservation considerably affected success of cryopreservation (Wang and Perl 2006). Using vitrification cryopreservation for different ages of *Arabidopsis thaliana* seedlings, Ren et al. (2013) found that survival of cryopreserved seedlings significantly decreased with an increase in age of stock seedlings: 97% and 0% for 48-h and 72-h seedlings, respectively. MDA was much higher in the 72-h stock seedlings than in the 48-h ones following osmoprotection and dehydration. There was a significant negative correlation between MDA content and survival levels following cryopreservation. A further study of the same group found that during the whole vitrification procedure, O_2_⋅− activities were much higher and H_2_O_2_ levels were much lower in the 48-h seedlings than in the 72-h ones. Chen et al. (2015) reported that although OH⋅ activities continuously increased during the whole cryopreservation procedure, the increased OH⋅ activities were much more pronounced in 72-h seedlings than in 48-h ones. These findings provide new insights on better understanding of the mechanism as to how the age of stock cultures influences plant cryopreservation.

Excision of the explant is a necessary step, particularly in cryopreservation of shoot tips and embryogenic tissues. Excision of the explant is a wounding process, which has been documented to induce accumulations of ROS (Savatin et al. 2014). Roach et al. (2008) reported that the excision of embryonic axes from the seeds of a recalcitrant sweet chestnut (*Castanea sativa*) induced an oxidative burst of O_2_⋅−, resulting in the reduced viability of the axes following desiccation and subsequent freezing in LN. More examples that explant excision-mediated oxidative stress were responsible for the reduced recoveries of cryopreserved explants are listed in Table 1.

**Table 1:** Some examples of ROS, cell damage and antioxidants induced in plant cryopreservation

Plant species	Explant	Cryopreservation method	ROS, cell damage and antioxidants detected	Steps of cryoprocedure, in which ROS, cell damage and antioxidants were detected	Reference
*Agapanthus praecox*	Embryogenic callus	Vitrification	O_2_⋅−, H_2_O_2_ and OH⋅; MDA; SOD, POD, CAT, AsA and GSH	PC, DH, FTC, UL, PCR	Zhang et al. (2015)
			O_2_⋅−, H_2_O_2_ and OH⋅; MDA; SOD, POD, CAT, AsA and GSH	PC, DH, FTC, UL, PCR	Chen et al. (2016)
			O_2_⋅−, H_2_O_2_ and OH⋅; MDA; SOD, POD, CAT, AsA and GSH	PC, DH, FTC, UL	Ren et al. (2020a)
			H_2_O_2_ and OH⋅; MDA; SOD, POD and CAT	PC, DH, UL, PCR	Chen et al. (2021a)
			O_2_⋅− and H_2_O_2_	DH, UL, PCR	Chen et al. (2021b)
*Amaryllis belladonna* and *Haemanthus montanus*	Zygotic embryos	Vitrification	O_2_⋅−; MDA; SOD, CAT, GR and APX	DH, FTC	Sershen et al. (2012)
*Arabidopsis thaliana*	Seedlings	Vitrification	MDA	DH	Ren et al. (2013)
			O_2_⋅−, H_2_O_2_ and OH⋅; MDA; SOD, POD, CAT, AsA and GSH	OP, DH, FTC, UL, PCR	Ren et al. (2015)
			O_2_⋅−, H_2_O_2_ and OH⋅; MDA; SOD, POD, CAT, AsA and GSH	OP, DH, FTC, UL, PCR	Chen et al. (2015)
			H_2_O_2_; MDA; CAT, AsA and GSH	OP, DH, FTC	Zhang et al. (2021a)
*Dendrobium nobile*	Protocorm-like bodies	Vitrification	O_2_⋅− and H_2_O_2_; MDA and PCO; SOD, CAT and AsA	PC, OP, DH, FTC, UL	Jia et al. (2016)
			H_2_O_2_	PC, OP, DH, FTC, UL	Jiang et al. (2019)
			H_2_O_2_ and NADPH; SOD, CAT, APX, GR, AsA and GSH	PC, OP, UL	Zhang et al. (2021b)
*Ekebergia capensis*	Seeds	Vitrification	O_2_⋅− and H_2_O_2_; total aqueous antioxidant (types not specified)	DH, FTC	Bharuth and Naidoo (2020)
*Eucalyptus grandis*	Axillary buds	Dehydration	O_2_⋅−	EE, PC, RE	Risenga et al. (2013)
*Hypericum perforatum*	Shoot tips	Vitrification	ROS and H_2_O_2_; SOD and CAT	EE, DH, PCR	Skyba et al. (2012)
*Lilium* × *siberia*	Pollen	Vitrification	ROS (types not specified)	OP, DH, FTC, UL	Xu et al. (2014)
*Magnolia denudata* and *Paeonia lactiflora*	Pollen	Direct immersion in LN	ROS (types not specified); MDA; SOD, CAT and AsA	FTC	Jia et al. (2018)
*Malus sylvestris*, *Prunus avium* and *Prunus padus*	Seeds	Dehydration	H_2_O_2_; MDA; AsA	PCR	Wawrzyniak et al. (2020)
*Oryza sativa*	Zygotic embryos	Vitrification	O_2_⋅− and H_2_O_2_; MDA; SOD, CAT, APX, DHAR, MDHAR, GR, AsA and GSH	PC, OP, DH	Huang et al. (2018)
*Paeonia lactifora*	Pollen	Direct immersion in LN	ROS (types not specified); MDA; SOD, CAT and AsA	FTC	Ren et al. (2019)
			H_2_O_2_ and OH⋅; SOD, POD, CAT, GR, APX, AsA and GSH	FTC	Ren et al. (2020b)
*Paeonia suffruticosa*	Pollen	Direct immersion in LN	O_2_⋅−, H_2_O_2_ and OH⋅; MDA and PCO; SOD, POD, CAT, GR, APX, AsA and GSH	FTC	Ren et al. (2021)
			ROS (types not specified)	FTC	Ren et al. (2020c)
*Paphiopedilum niveum*	Somatic embryos	Cryo-plate	ROS (types not specified); MDA	PC, DH	Soonthornkalump et al. (2020)
*Passiflora ligularis*	Seed embryos	Vitrification	H_2_O_2_; MDA; SOD, CAT and APX	PCR	Prudente et al. (2019)
*Strychnos gerrardii* and *Boophane disticha*	Zygotic axes	Dehydration	O_2_⋅−; total antioxidant activity	EE, DH, RH	Berjak et al. (2011)
*Trichilia dregeana*	Embryonic axes	Dehydration	O_2_⋅−; POD	EE, RH	Whitaker et al. (2010)
*Amygdalus davidiana*, *Kerria japonica*, *Jasminum nudiflorum*, *Orychophragmus violaceus* and *Paulownia tomentosa*	Pollen	Direct immersion in LN	ROS (types not specified)	FTC	Jia et al. (2017)
*Cercis chinensis*, *Magnolia biondii*, *Robinia pseudoacacia* and *Rosa primula*	Pollen	Direct immersion in LN	MDA	FTC	
*Malus spectabilis*, *Paeonia suffruticosa*, *Philadelphus pekinensis*, *Syringa oblata* and *Xanthoceras sorbifolium*	Pollen	Direct immersion in LN	ROS (types not specified) and MDA	FTC	

*LN* liquid nitrogen, *APX* ascorbate peroxidase, *AsA* ascorbic acid, *CAT* catalase, *DHAR* dehydroascorbate reductase, *GR* glutathione reductase, *GSH* glutathione, *H*_*2*_*O*_*2*_ hydrogen peroxide, *MDA* malondialdehyde, *MDHAR* monodehydroascorbate reductase, *NADPH* nicotinamide adenine dinucleotide phosphate, *O*_*2*_*⋅−* superoxide anion, *OH⋅* hydroxyl radicals, *PCO* protein carbonyl, *POD* peroxidase, *ROS* reactive oxygen species, *SOD* superoxide dismutase, *DH* dehydration, *EE* explant excision, *FTC* freeze–thaw cycle, *OP* osmoprotection, *PC* preculture, *PCR* post-culture for recovery, *DH* rehydration, *UL* unloading

Using a vitrification cryo-plate (V cryo-plate) for cryopreservation of *Paphiopedilum niveum* somatic embryos, Soonthornkalump et al. (2020) found that the highest level of the total ROS was induced in the preculture step. MDA level started to significantly increase from the preculture and reached the highest level in the osmoprotection step. These results indicated that preculture step was a critical step for successful cryopreservation of *Paphiopedilum niveum* somatic embryos.

Working on dehydration cryopreservation of recalcitrant seeds of *Ekebergia capensis*, Bharuth and Naidoo (2020) found that the reduced survival levels were closely associated with the increased levels of O_2_⋅− and H_2_O_2_ in each step of explant excision, cryoprotection, dehydration and freezing in LN. In the study of vitrification cryopreservation for *Oryza sativa* zygotic embryos, Huang et al. (2018) found that the major steps significantly affected levels of O_2_⋅− and MDA, which were negatively correlated with survival of the embryos following cryopreservation. Analyzing ROS production in zygotic embryos of recalcitrant *Haemanthus montanus* seeds subjected to cryopreservation, Sershen et al. (2012) found that although dehydration and cryoprotection increased O_2_⋅− levels, freezing in LN induced the highest level of O_2_⋅−, and was responsible for considerable or complete viability loss of the zygotic embryos following cryopreservation.

In vitrification cryopreservation of *Arabidopsis* seedlings, H_2_O_2_ and OH⋅ content continuously increased during the cryopreservation procedure and reached the highest level after rapid warming (Ren et al. 2015). Further analysis found that a large amount of ROS induced in the freeze–thaw cycle altered the redox state in the cell, caused membrane lipid peroxidation, and destroyed plant photosynthetic phosphorylation and oxidative phosphorylation systems (Ren et al. 2013, 2015). All these negative effects of excessive accumulations of ROS resulted in the reduced recovery of cryopreserved *Arabidopsis* seedlings.

It is worth noting that the production of ROS was found to increase the viability of pollen after cryopreservation in *Lilium* × *siberia* (Xu et al. 2014). In this study, ROS generation was analyzed by a flow cytometry with 2′,7′-dichlorodihydrofluorescein diacetate (DCFH-DA) fluorescent probe. The fluorescence levels at 488 nm (excitation) and 525 nm (emission) were expressed as mean green intensity fluorescence units for index of ROS level. ROS increased from 16 in the control to 26 and 50 in cryopreserved pollen by rapid cooling and vitrification, respectively. Pollen viability significantly increased from 47% in the control (fresh pollen) to 59% and 70% in cryopreserved pollen by rapid cooling and vitrification, respectively (Xu et al. 2014). These results indicate that ROS may have positive effects on cryopreservation, and the positive effects may depend on its concentration, plant species, types of explant and cryogenic procedures used.

ROS-induced oxidative stress and cell damage are illustrated in Fig. 1C, F and some examples of ROS generation and cell damage induced in plant cryogenic procedures are listed in Table 1.

### ROS-induced PCD

PCD refers to an ordered and energy-required physiological process that is genetically programmed, eventually leading to the cell death (Reape and McCabe 2008). There are three main forms in PCD, including apoptosis, necrosis and autophagic cell death (Reape and McCabe 2008). It has been well documented that the generation and accumulation of ROS under oxidative stress induced PCD, and apoptosis and necrosis were considered the major forms of PCD in cryopreservation (Reape and McCabe 2008; Bissoyi et al. 2014).

Harding et al. (2009) were the first to propose that cryopreservation may induce PCD in plants. Since then, oxidative stress-mediated ROS has been proven to be involved in induction of PCD in plant cryopreservation. Working on *Eucalyptus grandis* axillary buds, Risenga et al. (2013) found that bud excision and drying induced high ROS levels, which reduced viability of the explant analyzed by caspase-3-like protease activity. Caspases have been demonstrated to be responsible for the stress-triggered PCD processes (Reape and McCabe 2008). Therefore, Risenga et al. (2013) believed that dehydration-induced ROS generation triggered PCD. Wesley-Smith et al. (2015) found that exposure to LN resulted in autophagic degradation and ultimately autolysis, and formation of small intracellular ice crystals in cryopreserved embryonic axes of recalcitrant *Acer saccharinum* seeds. These results indicated that freezing stress induced PCD. In cryopreservation of *Dendrobium* PLBs by vitrification, Jiang et al. (2019) found that the preculture and freeze–thaw cycle induced expression of the autophagy-related protein 8C gene (*Atg 8C*) and reticulon-like protein B8 gene (*Rtnl B8*). Caspase-3-like activity increased following the osmoprotection treatment and dehydration and reached the highest level following freeze–thaw cycle. Levels of H_2_O_2_ and NO started to increase in the preculture stage and reached the highest level in the osmoprotection treatment. These results provided experimental concrete evidence that ROS generations triggered PCD in cryopreservation (Jiang et al. 2019).

Fluorescein diacetate (FDA) staining is generally used as a marker for apoptosis-like events (Burbridge et al. 2006). Ren et al. (2013) found that the 72-h *Arabidopsis* seedlings completely failed to recover following cryopreservation, compared with 97% survivals in the 48-h seedlings. The 72-h seedlings showed negative responses to FDA staining during the whole cryopreservation procedure. The defender against apoptotic death 1 (*DAD1*), which encodes for the protein involved in suppression of apoptosis (Gallois et al. 1997), was found to be upregulated in the 48-h seedlings, while its expression levels maintained unchanged in the 72-h ones after dehydration (Ren et al. 2013). These results supported that apoptosis-like events were induced in the 72-h *Arabidopsis* seedlings during cryopreservation.

In cryopreservation of *Agapanthus praecox* embryogenic callus (EC), Zhang et al. (2015) found that the burst outbreak of ROS induced the occurrence of PCD. Further analysis of PCD showed that some EC cells exhibited autophagy and apoptosis-like events, and a few cells underwent necrosis during cryopreservation. Autophagy appeared at the dehydration and unloading steps, and apoptosis-like events occurred through the cryopreservation procedure. Exogenous applications of PCD modulators like caspase inhibitor and cinnamtannin B-1 significantly improved cell viability following cryopreservation (Zhang et al. 2015).

In addition, ROS-triggered PCD has also been reported in cryopreservations of pollen of *Paeonia lactiflora* (Ren et al. 2020b), and *Paeonia suffruticosa* (Ren et al. 2020c), and *Agapanthus praecox* embryogenic callus (Chen et al. 2021a, b). ROS-induced PCD is illustrated in Fig. 1F.

### Adaptive enzymatic and non-enzymatic responses of antioxidant system

For enzymatic antioxidants, dehydration and freezing in LN were reported to reduce GR activity and aggravate cell membrane lipid peroxidation in zygotic embryos of *Zea mays* (Wen et al. 2010) and *Livistona chinensis* (Wen et al. 2012). Manipulations of pretreatments enhanced activities of antioxidants, especially GR, and partly increased tolerance to dehydration and freezing in LN of *Olea europaea* somatic embryos, resulting in increased survivals following cryopreservation (Lynch et al. 2011). Using droplet-vitrification cryopreservation of *Lomandra sonderi* shoot tips, Funnekotter et al. (2016) found that SOD activity was positively correlated to the recovery following cryopreservation, among the other three antioxidant enzymes CAT, GR and GPX.

In vitrification cryopreservation of *Dendrobium* sonia-28 protocorm-like bodies (PLBs), Poobathy et al. (2013) reported that drastic increases in CAT activities were produced following the treatments of preculture, thawing and unloading, and in the initial stage of shoot regrowth following cryopreservation. In the study of droplet-vitrification for cryopreservation of *Brassidium* PLBs, Rahmah et al. (2015) found that the activity of APX and CAT reached the highest level in dehydration and freezing in LN. SOD activities maintained an overall increase in different steps of the cryopreservation, and reached the highest level in the late shoot regrowth stage (Poobathy et al. 2013). Huang et al. (2018) showed that the preculture treatment stimulated O_2_⋅− generation and activated the antioxidative response system in the apical meristems of germinated embryos. Treatment of osmoprotection motivated the activity of CAT and APX. In the study on vitrification cryopreservation of *Dendrobium* Sabin Blue PLBs, Antony et al. (2019) found that the lowest activity of CAT was obtained in the explant after exposure to the PVS2 dehydration; the lowest activity of POX enzyme following the dehydration and post-culture for shoot regrowth; and the lowest activity of APX in the thaw and unloading steps. These changes in antioxidant enzymes resulted in the low level of shoot regrowth in the explant following cryopreservation (Antony et al. 2019). In a V cryo-plate cryopreservation for *Passifora suberosa* shoot tips, Vianna et al. (2019) reported that the highest SOD activity was detected in the preculture step, and continuously decreased during the cryopreservation procedure. Significantly higher activities of CAT and APX were obtained after the osmoprotection treatment. CAT activity gradually decreased after osmoprotection, whereas the APX activity decreased after 10 days of post-culture. All these results indicated that the elevated activities of enzymatic antioxidants enhanced cell resistance/tolerance to cryoinjury, and control of oxidative damage via ROS homeostasis can lead to the high recovery in cryopreservation.

For non-enzymatic antioxidants, Chen et al. (2015) found that the AsA content of 48-h *Arabidopsis* seedlings was significantly higher than in 72-h ones from the dehydration step to post-culture for recovery. Exogenous application of AsA or SK_3_-type dehydrin protein to PVS2 significantly elevated the content of endogenous AsA in the dehydration step, and increased the regrowth levels of *Arabidopsis* seedlings after cryopreservation (Ren et al. 2015; Zhang et al. 2021a). Significantly higher contents of AsA and GSH were induced in *A. praecox* EC following treatments of unloading and rewarming (Zhang et al. 2015). Chen et al. (2016) found that adding exogenous GSH to PVS2 increased endogenous AsA and GSH contents after dehydration step and improved cell viability following cryopreservation. Improved viability of cryopreserved pollen of *Paeonia suffruticosa* was attributed to sufficient maintenance of the internal balance of oxidative metabolism by SOD, AsA and GSH (Ren et al. 2021).

A recent study clearly demonstrated that the activities of antioxidants were closely related to success of cryopreservation (Ren et al. 2021). In this study, pollen viability following cryopreservation differed among three *P. sufruticosa* cultivars: one decreased, one was stable and one increased. Contents of ROS, MDA and protein carbonyl (PCO) were significantly lower in the cultivar with increased viability than in the other two cultivars, while SOD activity was higher in the former than in the latter two. SOD activity was negatively correlated with the MDA and POD contents, and positively correlated with pollen viability following cryopreservation. AsA was lower, while GSH was higher, in the cultivar with increased viability than in the other two cultivars. AsA significantly increased, while GSH significantly decreased, the MDA and PCO contents. The membrane lipid oxidation and protein oxidative damage caused by ROS were responsible for the decrease in pollen viability after cryopreservation. SOD, AsA and GSH effectively maintained the internal balance of oxidative metabolism and reduced the levels of oxidative damage, thus improving pollen viability (Ren et al. 2021).

Some examples are presented in Table 1 and adaptive responses of antioxidant system induced in plant cryopreservation are illustrated in Fig. 1G.

## Expressions of oxidative stress-related genes and proteins in plant cryopreservation

Over the past decade, efforts have been invested to study expressions of oxidative stress-related genes and proteins in plant cryopreservation. Some examples are presented in Table 2, and alleviation of ROS-induced oxidative stress by expressions of oxidative stress-related genes and proteins in plant cryopreservation is illustrated in Fig. 1G.

**Table 2:** Expressions of oxidative stress-related genes and proteins induced in plant cryopreservation

Plant species	Explant	Cryopreservation method	Major molecular experiments	Differentially expressed genes, proteins or pathways	Reference
*Agapanthus praecox*	Embryogenic callus	Vitrification	qRT-PCR	Eight oxidative stress related-genes and seven PCD related-genes	Zhang et al. (2015)
				Eight oxidative stress related-genes and two PCD related-genes	Chen et al. (2016)
				Twelve oxidative stress related-genes	Ren et al. (2020a)
				Ten cell death related genes	Chen et al. (2021b)
*Arabidopsis thaliana*	Shoot tips	Vitrification	Fully sequenced *Arabidopsis* genome and readily available microarray slides	Genes involved in cold, desiccation and oxidation responses	Volk et al. (2011)
			Array gene expression, qRT-PCR	Transcripts related to abiotic stress, oxidation, and wounding	Gross et al. (2017)
	Seedlings	Vitrification	cDNA-AFLP, qRT-PCR	Genes involved in stress response, protein synthesis and metabolism, and metabolism and energy	Ren et al. (2013)
			Transcriptome microarray, qRT-PCR	Metabolism, photosynthesis, carbohydrate, cofactor and vitamin metabolism, especially *DREBs*/*CBFs*, *calcium-binding protein*, *OXI1*, *WRKY* and *MYB* family members	Ren et al. (2013)
*Dendrobium*	Protocorm-like bodies	Vitrification	qRT-PCR	Six PCD-regulating genes	Jiang et al. (2019)
*Dendrobium nobile*	Protocorm-like bodies	Vitrification	iTRAQ proteomic analysis, qRT-PCR	Protein synthesis, processing and degradation, production of ROS, energy production, signaling transduction, and membrane transport	Di et al. (2018)
*Oryza sativa*	Zygotic embryos	Vitrification	qRT-PCR	Twenty antioxidant enzyme genes	Huang et al. (2018)
*Panax ginseng*	Embryogenic callus	Vitrification	Two-dimensional electrophoresis, qRT-PCR	Proteins related to carbohydrate metabolism, stress response, oxidative metabolism, and carbohydrate metabolism, especially HSP and 14-3-3-like protein	Lei et al. (2021)
*Prunus mume*	Pollen	Direct immersion in LN	Two-dimensional electrophoresis	Some protein spots between 12.6–72.8 and 5.6–7.3 kDa	Zhang et al. (2012)
*Solanum tuberosum* and *S. commersonii*	Shoot tips	Droplet vitrification	Two-dimensional electrophoresis	Proteins related to carbon fixation and mechanisms, oxidative homeostasis	Folgado et al. (2014)

*LN* liquid nitrogen, *cDNA-AFLP* cDNA amplified fragment length polymorphism, *iTRAQ* isobaric tags for relative and absolute quantification, *qRT-PCR* quantitative reverse transcription-polymerase chain reaction, *DREBs/CBFs* dehydration-responsive element-binding proteins/C-repeat binding factors, *MYB* v-Myb avian myeloblastosis viral oncogene homolog, *PCD* programmed cell death, *HSP* heat shock proteins, *miRNAs* microRNAs, *OXI1* oxidative signal-inducible 1, *ROS* reactive oxygen species, *WRKY* tryptophan-arginine-lysine-tyrosine

Huang et al. (2018) reported expressions of antioxidant enzyme genes *Cu*/*Zn SOD*, *CAT1*, *APX7*, *GR2*, *GR3*, *MDHAR1* and *DHAR1* in *Oryza sativa* zygotic embryos in vitrification cryopreservation. The authors suggested that these genes might serve as potential indicators of oxidative stress-induced genes in cryopreservation. Zhang et al. (2015) found that expressions of genes, particularly SOD and BAG, were positively related with cell viability of *Agapanthus praecox* EC following cryopreservation. Using high-throughput omics technology to screen two dehydrins of *A. praecox* EC subjected to cryopreservation, Yang et al. (2019) reported that their expression levels were specifically upregulated at the transcription and protein levels. Working on *A. praecox* EC, Chen et al. (2016) reported that inclusion of 0.08 mM GSH in PVS2 markedly increased recovery of cryopreserved EC and enhanced expressions of stress-responsive genes, including *POD*, *APX*, *MDHAR* and *GPX* in the treated samples during cryopreservation procedures. Gene expression patterns provided molecular mechanisms that the application of GSH to PVS2 solution effectively improved recovery of *A. praecox* EC.

Volk et al. (2011) used the *Arabidopsis* genome chip to analyze the differential expression genes of shoot tips during cryopreservation and found that dehydration-related genes were specifically upregulated after vitrification solution treatment. Application of 1 mM AsA in PVS2 significantly increased recovery in 60-h *Arabidopsis* seedlings following cryopreservation (Ren et al. 2014). DREBs/CBFs assisted establishments of tolerance cryoinjury, and calcium-binding protein, OXI1, WRKY and MYB family members served as key factors in ROS signal transduction and activated the ROS-producing and -scavenging networks. Increased expressions of these genes contributed to improvements of recovery of the 60-h seedlings following cryopreservation (Ren et al. 2015). Further studies by Chen et al. (2015) and Zhang et al. (2021a) both found that upregulations of *Cu*/*Zn*-*SOD*, *APX* and *CAT* increased tolerance to oxidative stress and improved the recovery of *Arabidopsis* seedlings after cryopreservation.

PVS2 and plant vitrification solution 3 (PVS3) are the most frequently used plant vitrification solutions in plant cryopreservation. Gross et al. (2017) found that expression levels of 180 transcripts differed in *Arabidopsis* shoot tips following exposures to PVS2 and PVS3. The transcripts induced by the treatment of PVS2 dehydration and freezing in LN induced oxidative responses, whereas the treatment of PVS3 dehydration and freezing in LN invoked more metabolic responses. These results provided molecular insights into varying levels of post-cryopreservation recoveries produced by PVS2 and PVS3 dehydration.

In the study of the translation level following treatments of sucrose or cold preculture of *Solanum tuberosum* and *S. commersonii* shoot tips, Folgado et al. (2014) found that the oxidative homeostasis-related proteins were associated with the improved tolerance to cryopreservation. In the study of cryopreservation of *Dendrobium nobile* PLBs, Di et al. (2018) found that protein synthesis, processing and degradation might be the main strategies to re-establish cell balance in the PLBs following cryopreservation. The production of ROS and the decline in energy production, signaling transduction, and membrane transport during LN exposure might be responsible for the viability loss. Studying microRNA (miRNA)-based post-transcriptional regulations in 48-h and 72-h *Arabidopsis* seedlings subjected to cryopreservation, Ekinci et al. (2021) demonstrated that the alteration of expression levels of cold-induced genes related-miRNAs played a key role in successful cryopreservation.

## Exogenous applications of antioxidants to alleviate oxidative stress for improving plant cryopreservation

A number of studies have been conducted over the past decade on exogenous applications of enzymatic and non-enzymatic antioxidants to alleviate oxidative stress for improving plant cryopreservation. Some examples are presented in Table 3. Improvement of recovery of cryopreserved plants by exogenous applications of the antioxidants is illustrated in Fig. 1D, G.

**Table 3:** Exogenous applications of enzymatic and non-enzymatic antioxidants for improving recovery of cryopreserved plants

Plant species	Explant	Cryopreservation method	Antioxidants (concentrations), application steps	Increases in recovery from control to treatment (survival, regrowth or germination)	Reference
Enzymatic antioxidants					
*Dendrobium nobile*	Protocorm-like bodies	Vitrification	CAT (400 U ml^−1^), UL	From 6 to 21% (regrowth)	Di et al. (2017)
			ETH (200–400 mg l^−1^), PC	From 67 to 73% (survival)	Zhang et al. (2021b)
*Euonymus fortunei*	Shoot tips	Vitrification	CAT (200 U ml^−1^), UL	From 27 to 58% (survival)	Xu et al. (2017)
*Magnolia denudata* and *Paeonia lactiflora*	Pollen	Direct immersion in LN	CAT (400 IU ml^−1^) and MDH (100 IU ml^−1^), FTC	From 24–46 to 64–70% for CAT and 30–84% for MDH (germination)	Jia et al. (2018)
Non-enzymatic antioxidants					
*Actinidia chinensis* var*. chinensis*	Shoot tips	Vitrification	AsA (0.4 mM), PT	From 0 to 40% (regrowth)	Mathew et al. (2019)
*Agapanthus praecox*	Embryogenic callus	Vitrification	Cinnamtannin B-1 (50 μg ml^−1^), DH	From 49.14 to 86.85% (survival)	Zhang et al. (2015)
			GSH (0.08 mM), DH	From 49 to 83% (survival)	Chen et al. (2016)
			Nanomaterials SWCNTs (0.1 g l^−1^), DH	From 53 to 85% (survival)	Ren et al. (2020a)
			ApSerpin-ZX protein (1.2 mg l^−1^), DH	From 33 to 70% (survival)	Chen et al. (2021a)
	Callus	Vitrification	Nanomaterials SWCNTs (0.1 g l^−1^) or C_60_ (0.3 g l^−1^), DH	From 24 to 49% for SWCNTs and 61% for C_60_ (survival)	Chen et al. (2017)
*Amaryllis belladonna*	Zygotic embryos	Dehydration	Glycerol (5–10%), PT	From 8 to 20–72%	Sershen et al. (2012)
*Arabidopsis thaliana*	60-h seedlings	Vitrification	ABA (1 µM), GB (10 mM), GSH (0.16 mM), AsA (1 mM), DH	From 18–29 to more than 40% (regrowth)	Ren et al. (2014)
			ApDHN protein (2 μM), DH	From 23 to 50% (regrowth)	Yang et al. (2019)
			Y_2_SK_2_- and SK_3_‑type dehydrins (5 µM), DH	From 25 to 46% (Y_2_SK_2_) and 52% (SK_3_) (regrowth)	Zhang et al. (2021a)
*Chrysanthemum grandiflorum*	Shoot tips	Vitrification	AFP (500–1000 µg l^−1^), DH	From 55–80 to 65–95% (regrowth)	Jeon et al. (2015)
*Dioscorea alata* and *D. cayenensis*	Shoot tips	Desiccation	Melatonin (0.05 and 0.1 μM), DH	From 15 to 35% (regrowth)	Uchendu and Keller (2016)
*Eucalyptus grandis*	Axillary buds		ABA (5 mg l^−1^), PT	From 55 to 70% (survival)	Risenga et al. (2013)
*Haemanthus montanus*	Zygotic embryos	Dehydration	Glycerol (5–10%), PT	From 10 to 15–55%	Sershen et al. (2012)
*Hancornia speciosa*	Lateral buds	Vitrification	Proline (0.1–0.2 M), PC	From 11 to > 40% (regrowth)	Prudente et al. (2017)
*Hypericum perforatum*	Shoot tips	Vitrification	ABA (0.076 µM), PT	From 59 to 71% (regrowth)	Bruňáková et al. (2011)
*Hypericum perforatum* and *Nicotiana tabacum*		Encapsulation-vitrification and vitrification	Melatonin (0.1–0.5 µM), PC and PCR	From 50–63 to 80–100% (regrowth)	Uchendu et al. (2014)
*Lamprocapnos spectabilis*	Shoot tips	Encapsulation-vitrification	Gold nanoparticles (10 ppm), EnCap	From 51 to 70% (regrowth)	Kulus and Tymoszuk (2021)
*Nephelium ramboutan-ake*		Vitrification	AsA (0.28 mM), OP	From 0 to 3.3% (regrowth)	Chua and Normah (2011)
*Oncidium flexuosum*	Mature seed	Vitrification	PG (1%), DH	From 47 to 78% (germination)	Galdiano et al. (2013)
			Supercool X-1000 (1%), DH	from 47 to 59% (germination)	
*Paeonia lactiflora*	Pollen	Direct freezing	HSP70 (0.5–10 μg ml^−1^), FTC	From about 21–28 to 26–36% (germination)	Ren et al. (2019)
*Paphiopedilum insigne*	Protocorm	Encapsulation-vitrification	Glutathione (30 µM), PC and PCR	From 37 to 63% (regrowth)	Diengdoh et al. (2019)
*Paphiopedilum niveum*	Somatic embryos	V cryo-plate	AsA (0.1 mM), PT	From 9 to 39% (regrowth)	Soonthornkalump et al. (2020)
*Picea abies*	Embryogenic tissue	Vitrification	ABA (10 µM), PT and DH	From 20 to 54% (survival)	Hazubska-Przybył et al. (2013)
*Picea glauca* × *P. engelmannii* and *Pseudotsuga menziesii*	Immature somatic embryo	Direct immersion in LN	ABA (50 µM), PT	From 10–40 to 100% for spruce genotype ISP 11 (survival)	Kong and von Aderkas (2011)
*Rhodiola crenulata*	Callus	Vitrification	Melatonin (0.1 µM), PC	From 62 to 72% (survival)	Zhao et al. (2011)
*Rubus*	Shoot tips	Vitrification	Vitamin E (11 mM), AsA (0.28 mM), PT, OP, UL and PCR	From 40 to 70% (VE) and from 40 to 90% (AsA) (regrowth)	Uchendu et al. (2010a)
			LA (4–8 mM), GSH (0.16 mM) and GB (10 mM), PT, OP, UL and PCR	From 40–50 to > 80% (regrowth)	Uchendu et al. (2010b)
*Strychnos gerrardii*	Zygotic axes	Drying and direct freezing	Cathodic water, RD	From 6 to 70% (regrowth)	Berjak et al. (2011)
*Ulmus americana*	Shoot tips	Vitrification and encapsulation-vitrification	Melatonin (0.1 µM), PC and PCR	From 50–63 to 80–100% (regrowth)	Uchendu et al. (2013)
*Vitis* spp.		Droplet-vitrification	SA (0.1 mM), PT of stock cultures	From 0–13 to 7–45% (regrowth)	Pathirana et al. (2016)

*LN* liquid nitrogen, *ABA* abscisic acid, *AsA* ascorbic acid, *AFP* antifreezing protein, *C*_*60*_ spherical carbon nanomaterial, *CAT* catalase, *DH* dehydration, *EnCap* encapsulation, *ETH* ethephon, *FTC* freeze–thaw cycle, *GB* glycine betaine, *GSH* glutathione, *HSP* heat shock protein, *LA* lipoic acid, *MDH* malate dehydrogenase, *OP* osmoprotection, *PC* preculture, *PCR* post-culture for recovery, *PG* phloroglucinol, *PT* pretreatment, *RD* redehydration, *SA* salicylic acid, *SWCNTs* single walled carbon nanotubes, *UL* unloading

### Enzymatic antioxidants

Testing effects of application of CAT or PDH into osmoprotection solution, PVS2 and unloading solution on vitrification cryopreservation of *Dendrobium nobile* PLBs, Di et al. (2017) found that adding of 400 IU ml^−1^ CAT to the unloading solution produced about 50% of survival and 21% of shoot regrowth in cryopreserved PLBs, which were significantly higher than 17% and 6% produced in the control. Another study from the same group found that only adding of 400 IU ml^−1^ CAT to osmoprotection solution significantly improved survival of *Euonynus fortunei* shoot tips (Xu et al. 2017). Jia et al. (2018) found that inclusion of 400 IU ml^−1^ CAT in the germination solution produced much higher pollen germination rates of cryopreserved pollen than the control in *Magnolia denudata* and *Paeonia lactifora*. The addition of malate dehydrogenase (MDH) in the germination solution was also found to improve pollen germination following cryopreservation, but the optimal concentrations varied with plant species: 100 IU ml^−1^ for *Magnolia denudata* and 200 IU ml^−1^ for *Paeonia lactifora* (Jia et al. 2018). These results indicated that effects of the antioxidant enzymes on cryopreservation vary with types and concentrations of antioxidant enzymes, steps in which antioxidant enzymes are added, and plant species and explant types. Inclusion of CAT and 2-4-carboxyphenyl-4,4,5,5-tetramethylimidazoline-1-oxyl-3-oxide in the preculture medium was reported to significantly increase survival in *Dendrobium* PLBs following cryopreservation (Jiang et al. 2019).

Exogenous application of CAT significantly reduced H_2_O_2_ and MDA contents, increased AsA content and induced higher activity of endogenous CAT in the treated PLBs of *Dendrobium nobile* (Di et al. 2017). Jia et al. (2018) found that the use of MDH significantly reduced ROS and MDA levels, but increased SOD activity in cryopreserved pollen of *M. denudata.* Application of CAT significantly reduced levels of ROS and MDA, but increased activity of CAT and SOD in *P. lactifora*. These effects of CAT and MDH were believed to contribute to the improvement of recovery in cryopreserved explants (Di et al. 2017; Jia et al. 2018).

### Non-enzymatic antioxidants

Over the past decade, there have been increasing interests in exogenous applications of melatonin and nanoparticles to improvements of plant cryopreservation. Non-enzymatic antioxidants such as AsA, ABA and GSH were also used as usual in plant cryopreservation.

#### Melatonin

Melatonin (*N*-acetyl-5-methoxytryptamine) is an indolic compound, which is naturally biosynthesized in diverse plant species including annual and perennial species, and woody and herbaceous species (Nawaz et al. 2016). It has been well documented that melatonin is involved in regulations of all plant development processes and enhancements of resistance and/or tolerance of plants to abiotic and biotic stress.

Zhao et al. (2011) were the first to demonstrate that exogenous application of melatonin significantly improved survival of *Rhodiola crenulata* callus following vitrification cryopreservation. In this study, the callus was pretreated with 0.1 µM melatonin at 25 °C in the dark for 5 days, followed by osmoprotection, exposure to PVS2 and freezing in LN. The melatonin pretreatment resulted in significantly higher survival level (72%) than other treatments. The melatonin pretreatment was found to significantly reduce MDA levels and increase POD and CAT activities during the whole cryopreservation procedure. Uchendu et al. (2013) reported that exogenous application of melatonin improved recovery levels of cryopreserved shoot tips of American elm (*Ulmus americana*). In this study, adding 0.1–0.5 µM melatonin into preculture and recovery medium resulted in 100% shoot regrowth in both in vitro-grown shoot tips and dormant buds following PVS2-vitrification and encapsulation-vitrification. More examples of the improved recovery of cryopreserved plants by exogenous application of melatonin can be found in Table 3.

#### Nanoparticles

Nanoparticles are defined as atomic or molecular aggregates, with their size smaller than 100 nm in at least one dimension (Preetha and Balakrishnan 2017). Applying gold nanoparticles (AuNPs) to preculture medium, alginate solution and recovery medium, Kulus and Tymoszuk (2021) reported that 10 ppm AuNPs added into the alginate solution produced much higher recovery of *Lamprocapnos spectabilis* shoot tips following cryopreservation. AuNPs treatment significantly increased the activities of antioxidant enzyme in the LN-derived plantlets. Adding of AuNPs at 10–30 ppm in the preculture medium or at 30 ppm in the alginate solution increased SOD activity. Much higher APX activities were obtained when 10–30 ppm AuNPs were added to the recovery medium, while adding 30 ppm AuNPs in the recovery medium significantly increased CAT activity.

Chen et al. (2017) reported that application of 0.3 g l^−1^ fullerene (C_60_) as a cryoprotectant significantly improved survival of *Agapanthus praecox* callus following cryopreservation. Application of C_60_ significantly reduced the relative conductivity, the MDA content and ROS activity in the treated callus. This datum indicates that the use of C_60_ can alleviate oxidative stress-induced in cryopreservation by protecting the cell membrane from damage and preventing the membrane lipid peroxidation. In addition, single-wall carbon nanotubes (SWCNTs) were reported to improve cryopreservation of *Agapanthus praecox* EC (Ren et al. 2020a). SWCNTs (0.1 g l^−1^) added in PVS2 entered EC, and most of the SWCNTs moved out at the unloading step. Analysis of the antioxidant system and oxidative stress-related gene expression found that the AsA-GSH and GPX cycle were responsible for scavenging H_2_O_2_ produced in the control, but the CAT cycle was essential for scavenging H_2_O_2_ produced in the SWCNTs-treated EC, thus reducing levels of H_2_O_2_ and MDA. Adding of SWCNTs in PVS2 increased the antioxidant levels during dehydration, thus enhancing the resistance of the SWCNTs-treated EC to oxidative stress and improving recovery of the SWCNT-treated EC.

#### Abscisic acid (ABA)

ABA has been well demonstrated to increase the tolerance and/or resistance of plants to abiotic stress including freezing (Sah et al. 2016). Kong and Aderkas (2011) described a novel method for efficient cryopreservation of somatic embryogenic tissues (SETs) of interior spruce (*Picea glauca* × *engelmannii*) and Douglas-fir (*Pseudotsuga menziesii* subsp. *menziesii*). Pretreatment of SETs with 50 µM ABA at 5 °C for 4–8 weeks produced the highest rate of survival and all survivors retained their embryogenic ability to regenerate whole plantlets in cryopreserved SETs.

Hazubska-Przybył et al. (2013) found that adding 10 µM ABA to the preculture medium improved recovery and normal plantlet regeneration in the cryopreserved SETs of Norway spruce (*Picea abies*). Assessments of genetic stability by simple sequence repeat (SSR) did not find any polymorphic bands in the plantlets regenerated from ABA-assisted cryopreservation. These results indicate that ABA can be considered safe for use in plant cryopreservation in terms of genetic stability of cryostored plants. More examples of the improved recovery of cryopreserved plants by exogenous application of ABA can be found in Table 3.

It is worth noting that Edesi et al. (2020) reported that preculture of the explant with 2–4 mg l^−1^ ABA at room temperature significantly decreased shoot regrowth in cryopreserved buds of *Rubus humulifolius.* There existed a synergistic effect of cold and ABA treatment in many plant species (Vandenbussche and Proft 1998; Chang and Reed 2001). Therefore, ABA-preculture at room temperature failed to produce positive effects or even exerted negative effects on cryopreservation, as reported by Edesi et al. (2020).

#### Ascorbic acid (AsA)

AsA (vitamin C) is the most effective water-soluble antioxidant in plants and plays an important role in protecting plants against oxidative stress (Smirnoff 2000; Gill and Tuteja 2010). In the study of V cryo-plate cryopreservation of *Paphiopedilum niveum* SEs, adding 0.1 mM AsA into the preconditioning medium 1 day before the 1st preculture produced much higher recovery (39%) than that (8.5%) of the control (Soonthornkalump et al. 2020). AsA treatments considerably reduced ROS and MDA levels in the cryopreservation steps including 1st preculture, 2nd preculture, osmoprotection and dehydration. More examples of the improved recovery of cryopreserved plants by exogenous application of AsA are presented in Table 3.

More recently, Khor et al. (2020) reported that the inclusion of AsA (50–150 mg l^−1^) in the medium of four steps, including preculture, osmoprotection, PVS2 dehydration and post-culture, reduced the shoot regrowth percentage of cryopreserved *Aranda broga* PLBs. Similar results were also obtained in cryopreserved *Rubus* shoot tips (Uchendu et al. 2010a). High concentrations of the antioxidant disturb the redox balance and result in cellular disfunction (Bouayed and Bohn 2010). Application of AsA (2 and 8 mM) increased ROS contents and inhibited growth of *Arabidopsis* seedlings (Qian et al. 2014). These results indicated that AsA might also work as a stress factor. Therefore, beneficial effects of antioxidants including AsA on cryopreservation are dose- and plant species-specific.

#### Glutathione (GSH)

GSH is one of the most abundant low molecular weight thiols, which is naturally biosynthesized in plants. Uchendu et al. (2010b) reported that exogenous application of GSH exerted positive effects on recovery of cryopreserved shoot tips of *Rubus.* Optimal GSH concentrations for the best results of cryopreservation varied with different steps in which GHS was added. Diengdoh et al. (2019) reported that adding of GSH in the preculture and recovery medium promoted recovery of *Paphiopedilum insigne* protocorms in vitrification and encapsulation-vitrification cryopreservation. In vitrification cryopreservation, 10–40 μM GSH enhanced shoot regrowth, with the best results obtained at 20 μM GSH; in encapsulation-vitrification, 10–50 μM GSH enhanced shoot regrowth, with the highest shoot regrowth obtained at 30 μM GSH.

Inclusion of 8 μM GSH in the cryoprotectant solution significantly improved survival of *Agapanthus praecox* EC (Chen et al. 2016). The application of GSH was found to reduce OH⋅ and O_2_⋅− production, as well as H_2_O_2_ and MDA contents, while increasing endogenous AsA and GSH contents in PVS2-dehydrated EC. GSH-treatment was also found to promote expression of oxidative stress-responsive genes, including *POD*, *APX*, *MDHAR* and *GPX* during cryopreservation processes, and enhance the expression of *DAD1*, a defender against apoptotic cell death, while suppressing cell death-related protease SBT. All these changes contributed to improvements of cryopreservation of *A. praecox* EC.

#### Others

Several other non-enzymatic antioxidants were reported to improve cryopreservation (Table 3), such as lipoic acid (LA), vitamin E and glycine betaine (GB) for *Rubus* shoot tips (Uchendu et al. 2010b), cathodic water for *Strychnos gerrardii* zygotic embryonic axes (Berjak et al. 2011), glycerol for zygotic embryos of *Amaryllis belladonna* and *Haemanthus montanus* (Sershen et al. 2012), phloroglucinol (PG) for *Oncidium flexuosum* seeds (Galdiano et al. 2013) and *Paphiopedilum insigne* protocorms (Diengdoh et al. 2019), salicylic acid for *Vitis* buds (Pathirana et al. 2016), Supercool X-1000^R^ for *Oncidium flexuosum* seeds (Galdiano et al. 2013), antifreeze protein (AFP) for *Chrysanthemum grandiflflorum* shoot tips (Jeon et al. 2015), proline for *Hancornia speciosa* buds (Prudente et al. 2017), ApSerpin-ZX protein for *Agapanthus praecox* embryogenic callus (Chen et al. 2021a), heat shock protein (HSP) for *Paeonia lactifora* pollen (Ren et al. 2019), and the LEA family recombinant dehydrin *(*DHN) proteins for *Arabidopsis thaliana* seedlings (Zhang et al. 2021a).

## Conclusion and perspectives

A number of the studies conducted in the past decade have proven that ROS unavoidably generated in the cryopreservation process and was responsible for the low success or even total failure of plant cryopreservation. ROS generation was identified to be the trigger for PCD. Adaptive responses of the antioxidant system and expressions of the oxidative stress-associated genes and proteins helped the plant to establish resistance and/or tolerance to the oxidative stress induced during cryopreservation. Applications of enzymatic and non-enzymatic antioxidants considerably improved recovery of various explants in different cryopreservation procedures and thus provided alternative strategies for efficient cryopreservation of plants. All these results are helpful for further developments and wider applications of plant cryobiotechnology.

Oxidative stress has been identified to be a major constraint for further developments of plant cryopreservation. To alleviate oxidative stress for improving recovery of cryopreserved plants, further studies should be strengthened in the following aspects: (1) to better understand how the explants respond to ROS-induced oxidative stress; (2) to elucidate the mechanism as to how the explants establish resistance and/or tolerance to ROS-induced oxidative stress; and (3) to apply both enzymatic and non-enzymatic antioxidants for improving cryopreservation of plants, particularly endangered, endemic and tropical species, which are still recalcitrant to cryopreservation; (4) various methods for quantitative analysis and visual observations have been used on the studies of ROS generations in plant cryopreservation. Novel and advanced techniques should be considered for use in the said subject. For example, fluorescence probes for monitoring ROS generations offer a potentially powerful tool for studying the chemistry and biology of ROS with high spatial and temporal resolution (Choi et al. 2012). High-throughput omics technology can be used to identify functions of the specific genes and proteins in protecting the cells against ROS-induced oxidative stress (Yang et al. 2019). These studies are expected to develop robust cryopreservation protocols, which can be used as routine methods for setting-up cryobanks of genetic resources of diverse plant species, and facilitate wider applications of cryobiotechnology.

### *Author contribution statement*

LR: data collection and analysis, writing of original manuscript; preparation of Tables 1, 2 and 3, and manuscript revision; M-RW: data collection and analysis, preparation of Fig. 1 and manuscript revision; Q-CW: proposal of the present study, manuscript revision and financial supports.

## Data Availability

The datasets generated or analyzed during the current study are available from the corresponding author on reasonable request.

## References

[CR1] Antony JJJ, Zakaria S, Zakaria R, Ujang JA, Othman N, Subramaniam S (2019). Biochemical analyses of *Dendrobium* Sabin Blue PLBs during cryopreservation by vitrification. Physiol Mol Biol Plant.

[CR2] Bednarek PT, Orłowska R (2020). Plant tissue culture environment as a switch-key of (epi)genetic changes. Plant Cell Tissue Organ Cult.

[CR3] Benson EE, Bremner D, Fuller BJ, Lane N, Benson EE (2004). Oxidative stress in the frozen plant: a free radical point of view. Life in the frozen state.

[CR4] Berjak P, Sershen VB, Pammenter NW (2011). Cathodic amelioration of the adverse effects of oxidative stress accompanying procedures necessary for cryopreservation of embryonic axes of recalcitrant-seeded species. Seed Sci Res.

[CR5] Bettoni JC, Bonnart R, Volk GM (2021). Challenges in implementing plant shoot tip cryopreservation technologies. Plant Cell Tissue Organ Cult.

[CR6] Bharuth V, Naidoo C (2020). Responses to cryopreservation of recalcitrant seeds of *Ekebergia capensis* from different provenances. S Afr J Bot.

[CR7] Bi W, Saxena A, Ayyanath MM, Harpur C, Shukla MR, Saxena PK (2021). Conservation, propagation, and redistribution (CPR) of Hill’s thistle: paradigm for plant species at risk. Plant Cell Tissue Organ Cult.

[CR8] Bissoyi A, Nayak B, Pramanik K, Sarangi SK (2014). Targeting cryopreservation-induced cell death: a review. Biopreservation Biobanking.

[CR9] Bouayed J, Bohn T (2010). Exogenous antioxidants—double-edged swords in cellular redox state: health beneficial effects at physiologic doses versus deleterious effects at high doses. Oxid Med Cell Longev.

[CR10] Bruňáková K, Zámečník J, Urbanová M, Čellárová E (2011). Dehydration status of ABA-treated and cold-acclimated *Hypericum perforatum* L. shoot tips subjected to cryopreservation. Thermochim Acta.

[CR11] Burbridge E, Diamond M, Dix PJ, McCabe PF (2006). Use of cell morphology to evaluate the effect of a peroxidase gene on cell death induction thresholds in tobacco. Plant Sci.

[CR12] Chaitanya KV, Sundar D, Masilamani S, Ramachandra Reddy A (2002). Variation in heat stress-induced antioxidant enzyme activities among three mulberry cultivars. Plant Growth Regul.

[CR13] Chang Y, Reed BM (2001). Preculture conditions influence cold hardiness and regrowth of *Pyrus cordata* shoot tips after cryopreservation. HortScience.

[CR14] Chen GQ, Ren L, Zhang J, Reed BM, Zhang D, Shen XH (2015). Cryopreservation affects ROS-induced oxidative stress and antioxidant response in *Arabidopsis* seedlings. Cryobiology.

[CR15] Chen GQ, Ren L, Zhang D, Shen XH (2016). Glutathione improves survival of cryopreserved embryogenic calli of *Agapanthus praecox* subsp. *orientalis*. Acta Physiol Plant.

[CR16] Chen S-M, Ren L, Zhang D, Zhang Y-F, Shen X-H (2017). Carbon nanomaterials enhance survival rates of *Agapanthus praecox* callus after vitrification cryopreservation. CryoLetters.

[CR17] Chen G, Li R, Shen X (2021). ApSerpin-ZX from *Agapanthus praecox*, is a potential cryoprotective agent to plant cryopreservation. Cryobiology.

[CR18] Chen G, Zhang D, Pan J, Yue J, Shen X (2021). Cathepsin B-like cysteine protease ApCathB negatively regulates cryo-injury tolerance in transgenic *Arabidopsis* and *Agapanthus praecox*. Plant Sci.

[CR19] Choi W-G, Swanson SJ, Gilroy S (2012). High-resolution imaging of Ca_2_^+^, redox status, ROS and pH using GFP biosensors. Plant J.

[CR20] Chua SP, Normah MN (2011). Effect of preculture, PVS2 and vitamin C on survival of recalcitrant *Nephelium ramboutanake* shoot tips after cryopreservation by vitrification. CryoLetters.

[CR21] Coelho N, Gonçalves S, Romano A (2020). Endemic plant species conservation: biotechnological approaches. Plants.

[CR22] Di W, Jia M, Xu J, Li B, Liu Y (2017). Exogenous catalase and pyruvate dehydrogenase improve survival and regeneration and affect oxidative stress in cryopreserved *Dendrobium nobile* protocorm-like bodies. CryoLetters.

[CR23] Di W, Jiang X, Xu J, Jia M, Li B, Liu Y (2018). Stress and damage mechanisms in *Dendrobium nobile* Lindl. protocorm-like bodies during pre- and post-liquid nitrogen exposure in cryopreservation revealed by iTRAQ proteomic analysis. In Vitro Cell Dev Biol Plant.

[CR24] Diengdoh RV, Kumaria S, Das MC (2019). Antioxidants and improved regrowth procedure facilitated cryoconservation of *Paphiopedilum insigne* Wall. Ex. Lindl. - an endangered slipper orchid. Cryobiology.

[CR25] Edesi J, Tolonen J, Ruotsalainen AL, Aspi J, Häggman H (2020). Cryopreservation enables long-term conservation of critically endangered species *Rubus humulifolius*. Biodivers Conserv.

[CR26] Ekinci MH, Kayıhan DS, Kayıhan C, Çiftçi YÖ (2021). The role of microRNAs in recovery rates of *Arabidopsis thaliana* after short term cryo-storage. Plant Cell Tissue Organ Cult.

[CR27] Folgado R, Sergeant K, Renaut J, Swennen R, Hausman J-F, Panis B (2014). Changes in sugar content and proteome of potato in response to cold and dehydration stress and their implications for cryopreservation. J Proteomics.

[CR29] Funnekotter B, Sortey A, Bunn E, Turner S, Mancera R (2016). Influence of abiotic stress preconditioning on antioxidant enzymes in shoot tips of *Lomandra sonderi* (Asparagaceae) prior to cryostorage. Aust J Bot.

[CR30] Funnekotter B, Colville L, Kaczmarczyk A, Turner SR, Bunn E, Mancera RL (2017). Monitoring of oxidative status in three native Australian species during cold acclimation and cryopreservation. Plant Cell Rep.

[CR31] Galdiano RF, de Macedo Lemos EG, Vendrame WA (2013). Cryopreservation, early seedling development, and genetic stability of *Oncidium flexuosum* Sims. Plant Cell Tissue Organ Cult.

[CR32] Gallois P, Makishima T, Hecht V, Despres B, Laudie M, Nishimoto T, Cooke R (1997). An *Arabidopsis thaliana* cDNA complementing a hamster apoptosis suppressor mutant. Plant J.

[CR33] Gill SS, Tuteja N (2010). Reactive oxygen species and antioxidant machinery in abiotic stress tolerance in crop plants. Plant Physiol Biochem.

[CR34] Gross BL, Henk AD, Bonnart R, Volk GM (2017). Changes in transcript expression patterns as a result of cryoprotectant treatment and liquid nitrogen exposure in *Arabidopsis* shoot tips. Plant Cell Rep.

[CR35] Halder T, Upadhyaya G, Basak C, Das A, Chakraborty C, Ray S (2018). Dehydrins impart protection against oxidative stress in transgenic tobacco plants. Front Plant Sci.

[CR36] Halliwell B (2006). Reactive species and antioxidants. Plant Physiol.

[CR37] Harding K, Johnston JW, Benson EE (2009). Exploring the physiological basis of cryopreservation success and failure in clonally propagated *in vitro* crop plant germplasm. Agric Food Sci.

[CR38] Hazubska-Przybył T, Chmielarz P, Michalak M, Dering M, Bojarczuk K (2013). Survival and genetic stability of *Picea abies* embryogenic cultures after cryopreservation using a pregrowth-dehydration method. Plant Cell Tissue Organ Cult.

[CR39] Huang B, Zhang JM, Chen XL, Xin X, Yin GK, He JJ, Lu XX, Zhou YC (2018). Oxidative damage and antioxidantive indicators in 48 h germinated rice embryos during the vitrification-cryopreservation procedure. Plant Cell Rep.

[CR41] Jenderek MM, Reed BM (2017). Cryopreserved storage of clonal germplasm in the USDA National Plant Germplasm System. In Vitro Cell Dev Biol Plant.

[CR42] Jeon SM, Naing AH, Park KI, Kim CK (2015). The effect of antifreeze protein on the cryopreservation of chrysanthemums. Plant Cell Tissue Organ Cult.

[CR43] Jia MX, Di W, Liu Y, Shi Y, Xie Y (2016). ROS-induced oxidative stress in nobile-type *Dendrobium* protocorm-like bodies (PLBS) during vitrification. CryoLetters.

[CR44] Jia MX, Shi Y, Di W, Jiang XR, Xu J, Liu Y (2017). ROS-induced oxidative stress is closely related to pollen deterioration following cryopreservation. In Vitro Cell Dev Biol Plant.

[CR45] Jia MX, Jiang RJ, Xu J, Di W, Shi Y, Liu Y (2018). CAT and MDH improve the germination and alleviate the oxidative stress of cryopreserved *Paeonia* and *Magnolia* pollen. Acta Physiol Plant.

[CR46] Jiang X, Ren R, Di W, Jia M, Li Z, Liu Y, Gao R (2019). Hydrogen peroxide and nitric oxide are involved in programmed cell death induced by cryopreservation in *Dendrobium* protocorm-like bodies. Plant Cell Tissue Organ Cult.

[CR47] Kaczmarczyk A, Funnekotter B, Menon A, Phang PY, Al-Hanbali A, Bunn E, Mancera RL, Katkov II (2012). Current issues in plant cryopreservation. Current frontiers in cryobiology.

[CR48] Khor SP, Yeow LC, Poobathy R, Zakaria R, Chew BL, Subramaniam S (2020). Droplet-vitrification of *Aranda* Broga Blue orchid: role of ascorbic acid on the antioxidant system and genetic fidelity assessments via RAPD and SCoT markers. Biotechnol Rep.

[CR49] Kong L, von Aderkas P (2011). A novel method of cryopreservation without a cryoprotectant for immature somatic embryos of conifer. Plant Cell Tissue Organ Cult.

[CR50] Kulus D, Tymoszuk A (2021). Gold nanoparticles affect the cryopreservation efficiency of in vitro-derived shoot tips of bleeding heart. Plant Cell Tissue Organ Cult.

[CR51] Kulus D, Zalewska M (2014). Cryopreservation as a tool used in long-term storage of ornamental species—a review. Sci Hortic.

[CR52] Lei X, Wang Q, Yang H, Qi Y, Hao X, Wang Y (2021). Vitrification and proteomic analysis of embryogenic callus of *Panax ginseng* CA Meyer. In Vitro Cell Dev Biol Plant.

[CR53] Lynch PT, Siddika A, Johnston JW, Trigwell SM, Mehra A, Benelli C, Lambardi M, Benson EE (2011). Effects of osmotic pretreatments on oxidative stress, antioxidant profiles and cryopreservation of olive somatic embryos. Plant Sci.

[CR54] Mathew L, Burritt DJ, McLachlan A, Pathirana R (2019). Combined pre-treatments enhance antioxidant metabolism and improve survival of cryopreserved kiwifruit shoot tips. Plant Cell Tissue Organ Cult.

[CR56] Mittler R (2002). Oxidative stress, antioxidants and stress tolerance. Trend Plant Sci.

[CR57] Mittler R (2017). ROS are good. Trend Plant Sci.

[CR58] Nawaz MA, Huang Y, Bie Z, Ahmed W, Reiter RJ, Niu M, Hameed S (2016). Melatonin: current status and future perspectives in plant science. Front Plant Sci.

[CR59] Noctor G, Foyer CH (1998). Ascorbate and glutathione: keeping active oxygen under control. Ann Rev Plant Biol.

[CR60] Normah MN, Sulonga N, Reed BM (2019). Cryopreservation of shoot tips of recalcitrant and tropical species: advances and strategies. Cryobiology.

[CR61] Pastori G, Mullineaux P, Foyer CH (2000). Post transcriptional regulation prevents accumulation of glutathione reductase protein and activity in the bundle sheath cells of maize. Implication on the sensitivity of maize to temperatures. Plant Physiol.

[CR62] Pathirana R, McLachlan A, Hedderley D, Panis B, Carimi F (2016). Pre-treatment with salicylic acid improves plant regeneration after cryopreservation of grapevine (*Vitis* spp.) by droplet vitrification. Acta Physiol Plant.

[CR63] Poobathy R, Sinniah UR, Xavier R, Subramaniam S (2013). Catalase and superoxide dismutase activities and the total protein content of protocorm-like bodies of *Dendrobium* Sonia-28 subjected to vitrification. Appl Biochem Biotechnol.

[CR64] Preetha PS, Balakrishnan N (2017). A review of nano fertilizers and their use and functions in soil. Int J Curr Microbiol Appl Sci.

[CR65] Prudente DO, Paiva R, Nery FC, Paiva PDO, Alves JD, Máximo WPF, Silva LC (2017). Compatible solutes improve regrowth, ameliorate enzymatic antioxidant systems, and reduce lipid peroxidation of cryopreserved *Hancornia speciosa* Gomes lateral buds. In Vitro Cell Dev Biol Plant.

[CR66] Prudente DO, Paiva R, Domiciano D, Souza LB, Carpentier S, Swennen R, Silva LC, Nery FC, Máximo WPF, Panis B (2019). The cryoprotectant PVS2 plays a crucial role in germinating *Passiflora ligularis* embryos after cryopreservation by influencing the mobilization of lipids and the antioxidant metabolism. J Plant Physiol.

[CR67] Qian HF, Peng XF, Han X, Ren J, Zhan KY, Zhu M (2014). The stress factor, exogenous ascorbic acid, affects plant growth and the antioxidant system in *Arabidopsis thaliana*. Russ J Plant Physiol.

[CR68] Rahmah S, Mubbarakh A, Ping S, Subramaniam KS (2015). Effects of droplet-vitrification cryopreservation based on physiological and antioxidant enzyme activities of *Brassidium* shooting star orchid. Sci World J.

[CR69] Reape TJ, McCabe P (2008). Apoptotic-like programmed cell death in plants. New Phytol.

[CR70] Reed BM (2014). Antioxidants and cryopreservation, the new normal?. Acta Hortic.

[CR71] Ren L, Zhang D, Jiang X, Gai Y, Wang W, Reed BM, Shen X (2013). Peroxidation due to cryoprotectant step is a vital factor for cell survival in *Arabidopsis* cryopreservation. Plant Sci.

[CR72] Ren L, Zhang D, Shen XH, Reed RM (2014). Antioxidants and anti-stress compounds improve the survival of cryopreserved *Arabidopsis* seedlings. Acta Hortic.

[CR73] Ren L, Zhang D, Chen G, Reed BM, Shen X, Chen H (2015). Transcriptomic profiling revealed the regulatory mechanism of *Arabidopsis* seedlings response to oxidative stress from cryopreservation. Plant Cell Rep.

[CR74] Ren R, Jiang X, Di W, Li Z, Li B, Xu J, Liu Y (2019). HSP70 improves the viability of cryopreserved *Paeonia lactiflora* pollen by regulating oxidative stress and apoptosis-like programmed cell death events. Plant Cell Tissue Organ Cult.

[CR75] Ren L, Deng S, Chu Y, Zhang Y, Zhao H, Chen H, Zhang D (2020). Single-wall carbon nanotubes improve cell survival rate and reduce oxidative injury in cryopreservation of *Agapanthus praecox* embryogenic callus. Plant Methods.

[CR76] Ren R, Li Z, Jiang X, Liu Y (2020). The ROS-associated programmed cell death causes the decline of pollen viability recovered from cryopreservation in *Paeonia lactiflora*. Plant Cell Rep.

[CR77] Ren R, Li Z, Zhou H, Zhang L, Jiang X, Liu Y (2020). Changes in apoptosis-like programmed cell death and viability during the cryopreservation of pollen from *Paeonia suffruticosa*. Plant Cell Tissue Organ Cult.

[CR78] Ren R, Li Z, Zhang L, Zhou H, Jiang X, Liu Y (2021). Enzymatic and nonenzymatic antioxidant systems impact the viability of cryopreserved *Paeonia suffruticosa* pollen. Plant Cell Tissue Organ Cult.

[CR79] Risenga I, Watt P, Mycock D (2013). Programmed cell death and necrosis during cryopreservative drying of *in vitro Eucalyptus grandis* axillary buds. CryoLetters.

[CR80] Roach T, Ivanova M, Beckett RP, Minibayeva FV, Green I, Pritchard HW, Kranner I (2008). An oxidative burst of superoxide in embryonic axes of recalcitrant sweet chestnut seeds as induced by excision and desiccation. Physiol Plant.

[CR81] Sah SK, Reddy KR, Li J (2016). Abscisic acid and abiotic stress tolerance in crop plants. Front Plant Sci.

[CR82] Salama A, Popova E, Jones MP, Shukla MR, Fisk NS, Saxena PK (2018). Cryopreservation of the critically endangered golden paintbrush (*Castilleja levisecta* Greenm.): from nature to cryobank to nature. In Vitro Cell Dev Biol Plant.

[CR83] Savatin DV, Gramegna G, Modesti V, Cervone F (2014). Wounding in the plant tissue: the defense of a dangerous passage. Front Plant Sci.

[CR84] Scandalios JG (1990). Response of plant antioxidant defense genes to environmental stress. Adv Gen.

[CR85] Sershen VB, Pammenter NW, Berjak P (2012). Cryo-tolerance of zygotic embryos from recalcitrant seeds in relation to oxidative stress—a case study on two amaryllid species. J Plant Physiol.

[CR86] Skyba M, Petijová L, Košuth J, Koleva DP, Ganeva TG, Kapchina-Toteva VM, Čellárová E (2012). Oxidative stress and antioxidant response in *Hypericum perforatum* L. plants subjected to low temperature treatment. J Plant Physiol.

[CR87] Smirnoff N (2000). Ascorbic acid: metabolism and functions of a multifacetted molecule. Curr Opin Plant Biol.

[CR88] Soonthornkalump S, Yamamoto S-i, Meesawat U (2020). Adding ascorbic acid to reduce oxidative stress during cryopreservation of somatic embryos of *aphiopedilum niveum* (Rchb.f.) Stein, an endangered orchid species. Hortic J.

[CR89] Streczynski R, Clark H, Whelehan LM, Ang S-T, Hardstaff LK, Funnekotter B, Bunn E, Offord CA, Sommerville KD, Mancera RL (2019). Current issues in plant cryopreservation and importance for *ex situ* conservation of threatened Australian native species. Aust J Bot.

[CR90] Uchendu EE, Keller ERJ (2016). Melatonin-laoded alginate beads improved cryopreservation of yam (*Dioscorea alata* and *D. cayenensis*). CryoLetters.

[CR91] Uchendu EE, Leonard SW, Traber MG, Reed BM (2010). Vitamins C and E improve regrowth and reduce lipid peroxidation of blackberry shoot tips following cryopreservation. Plant Cell Rep.

[CR92] Uchendu EE, Muminova M, Gupta S, Reed MM (2010). Antioxidant and anti-stress compounds improve regrowth of cryopreserved *Rubus* shoot tips. In Vitro Cell Dev Biol Plant.

[CR93] Uchendu EE, Shukla MR, Reed BM, Saxena PK (2013). Melatonin enhances the recovery of cryopreserved shoot tips of American elm (*Ulmus americana* L.). J Pineal Res.

[CR94] Uchendu EE, Shukla MR, Reed BM, Saxena PK (2014). An efficient method for cryopreservation of St John’s Wort and tobacco: role of melatonin. Acta Hortic.

[CR95] Vandenbussche B, De Proft MP (1998). Cryopreservation of in vitro sugar beet shoot tips using the encapsulation-dehydration technique: influence of abscisic acid and cold acclimation. Plant Cell Rep.

[CR96] Vianna MG, Garcia RO, Mansur E, Engelmann F, Pacheco G (2019). Oxidative stress during the cryopreservation of *Passiflora suberosa* L. shoot tips using the V-Cryo-plate technique: determination of the critical stages of the protocol. Plant Cell Tissue Organ Cult.

[CR97] Volk GM, Henk A, Basu C (2011). Gene expression in response to cryoprotectant and liquid nitrogen exposure in *Arabidopsis* shoot tips. Acta Hortic.

[CR98] Vollmer R, Villagaray R, Egúsquiza V, Espirilla J, García M, Torres A, Rojas E, Panta A, Barkley N, Ellis D (2016). The potato cryobank at the International Potato Center (CIP): a model for long term conservation of clonal plant genetic resources collections of the future. CryoLetters.

[CR99] Vranová E, Inzé D, Van Breusegem F (2002). Signal transduction during oxidative stress. J Exp Bot.

[CR100] Wang QC, Perl A, Silva JT (2006). Cryopreservation in floricultural crops. Floricultural, ornamental and plant biotechnology: advances and topics.

[CR101] Wang Q-C, Valkonen JPT (2009). Cryotherapy of shoot tips: novel pathogen eradication method. Trend Plant Sci.

[CR102] Wang B, Zhang Z, Yin Z, Feng C, Wang Q-C (2012). Novel and potential application of cryopreservation to plant genetic transformation. Biotechnol Adv.

[CR103] Wang B, Wang R-R, Cui Z-H, Bi W-L, Li J-W, Li B-Q, Ozudogru EA, Volk GM, Wang Q-C (2014). Potential applications of cryogenic technologies to plant genetic improvement and pathogen eradication. Biotechnol Adv.

[CR104] Wang M-R, Lambardi M, Engelmann F, Pathirana R, Panis B, Volk GM, Wang Q-C (2021). Advances in cryopreservation of *in vitro*-derived propagules: technologies and explant resources. Plant Cell Tissue Organ Cult.

[CR105] Wang M-R, Bi W, Shukla MR, Ren L, Hamborg Z, Blystad D-R, Saxena PK, Wang Q-C (2021). Epigenetic and genetic integrity, metabolic stability, and field performance of cryopreserved plants. Plants.

[CR106] Wawrzyniak MK, Michalak M, Chmielarz P (2020). Effect of different conditions of storage on seed viability and seedling growth of six European wild fruit woody plants. Ann for Sci.

[CR107] Wen B, Wang R, Cheng H, Song S (2010). Cytological and physiological changes in orthodox maize embryos during cryopreservation. Protoplasma.

[CR108] Wen B, Cai C, Wang R, Song S, Song J (2012). Cytological and physiological changes in recalcitrant Chinese fanpalm (*Livistona chinensis*) embryos during cryopreservation. Protoplasma.

[CR109] Wesley-Smith J, Walters C, Pammenter NW, Berjak P (2015). Why is intracellular ice lethal? A microscopical study showing evidence of programmed cell death in cryo-exposed embryonic axes of recalcitrant seeds of *Acer saccharinum*. Ann Bot.

[CR110] Whitaker C, Beckett RP, Minibayeva FV, Kranner I (2010). Production of reactive oxygen species in excised and cryopreserved explants of *Trichilia dregeana* Sond. S Afr J Bot.

[CR111] Xu J, Liu Q, Jia M, Liu Y, Li B, Shi Y (2014). Generation of reactive oxygen species during cryopreservation may improve *Lilium × siberia p*ollen viability. In Vitro Cell Dev Biol Plant.

[CR112] Xu J, Liu Y, Li B, Wang Z, Liu Q, Shi Y (2017). Effects of catalase and malate dehydrogenase on cryopreservation of *Euonymus forunei* (Turcz.) Hand.-Maz. Shoot tips by vitrification. Propag Ornam Plant.

[CR113] Yang Z, Sheng J, Lv K, Ren L, Zhang D (2019). Y_2_SK_2_ and SK_3_ type dehydrins from *Agapanthus praecox* can improve plant stress tolerance and act as multifunctional protectants. Plant Sci.

[CR114] Zhang YL, Li BL, Wang H, Liu Y (2012). Changes in total soluble proteins and Ca^2+^ upon cryopreservation of *Prunus mume* pollen. CryoLetters.

[CR115] Zhang D, Ren L, Chen GQ, Zhang J, Reed BM, Shen XH (2015). ROS-induced oxidative stress and apoptosis-like event directly affect the cell viability of cryopreserved embryogenic callus in *Agapanthus praecox*. Plant Cell Rep.

[CR116] Zhang D, Yang T, Ren L (2021). Y_2_SK_2_- and SK_3_-type dehydrins from *Agapanthus praecox* act as protectants to improve plant cell viability during cryopreservation. Plant Cell Tissue Organ Cult.

[CR117] Zhang L, Ren R, Jiang X, Zhou H, Liu Y (2021). Exogenous ethylene increases the viability of cryopreserved *Dendrobium* protocorm-like bodies by regulating the hydrogen peroxide and its mediated oxidative stress. Plant Cell Tissue Organ Cult.

[CR118] Zhao Y, Qi LW, Wang WM, Saxena PK, Liu CZ (2011). Melatonin improves the survival of cryopreserved callus of *Rhodiola crenulata*. J Pineal Res.

[CR119] Zhao L, Wang M-R, Li J-W, Volk GM, Wang Q-C (2019). Cryobiotechnology: a double-edged sword for plant obligate pathogens. Plant Dis.

